# Exhaled Volatile Organic Compounds Precedes Pulmonary Injury in a Swine Pulmonary Oxygen Toxicity Model

**DOI:** 10.3389/fphys.2019.01297

**Published:** 2019-12-03

**Authors:** William A. Cronin, Angela S. Forbes, Kari L. Wagner, Peter Kaplan, Renee Cataneo, Michael Phillips, Richard Mahon, Aaron Hall

**Affiliations:** ^1^Walter Reed National Military Medical Center, Bethesda, MD, United States; ^2^Undersea Medicine Department, Naval Medical Research Center, Silver Spring, MD, United States; ^3^Breath Research Laboratory, Menssana Research, Inc., Newark, NJ, United States; ^4^Henry M. Jackson Foundation for the Advancement of Military Medicine, Bethesda, MD, United States

**Keywords:** pulmonary oxygen toxicity, volatile organic compounds, hyperoxia, swine, prediction

## Abstract

**Purpose:**

Inspiring high partial pressure of oxygen (FiO_2_ > 0.6) for a prolonged duration can lead to lung damage termed pulmonary oxygen toxicity (PO_2_T). While current practice is to limit oxygen exposure, there are clinical and military scenarios where higher FiO_2_ levels and partial pressures of oxygen are required. The purpose of this study is to develop a non-invasive breath-based biomarker to detect PO_2_T prior to the onset of clinical symptoms.

**Methods:**

Male Yorkshire swine (20–30 kg) were placed into custom airtight runs and randomized to air (0.209 FiO_2_, *n* = 12) or oxygen (>0.95 FiO_2_, *n* = 10) for 72 h. Breath samples, arterial blood gases, and vital signs were assessed every 12 h. After 72 h of exposure, animals were euthanized and the lungs processed for histology and wet-dry ratios.

**Results:**

Swine exposed to hyperoxia developed pulmonary injury consistent with PO_2_T. Histology of oxygen-exposed swine showed pulmonary lymphatic congestion, epithelial sloughing, and neutrophil transmigration. Pulmonary injury was also evidenced by increased interstitial edema and a decreased PaO_2_/FiO_2_ ratio in the oxygen group when compared to the air control group. Breath volatile organic compound (VOC) sample analysis identified six VOCs that were combined into an algorithm which generated a breath score predicting PO_2_T with a ROC/AUC curve of 0.72 defined as a of PaO_2_/FiO_2_ ratio less than 350 mmHg.

**Conclusion:**

Exposing swine to 72 h of hyperoxia induced a pulmonary injury consistent with human clinical endpoints of PO_2_T. VOC analysis identified six VOCs in exhaled breath that preceded PO_2_T. Results show promise that a simple, non-invasive breath test could potentially predict the risk of pulmonary injury in humans exposed to high partial pressures of oxygen.

## Introduction

Inhaled oxygen administration is common in both healthcare settings and certain military operations for numerous indications. However, prolonged exposure to a fractional oxygen concentration > 0.6 is associated with significant pulmonary injury (Clark and Lambertsen, 1971; Jenkinson, 1993; Hedley-Whyte, 2008). To minimize oxygen toxicity in clinical settings, the fractional inspired oxygen (FiO_2_) is typically titrated to maintain a hemoglobin saturation of >90% (Beers, 2008; Mikkelsen et al., 2014); however, there are clinical scenarios such as acute respiratory distress syndrome (ARDS) and refractory hypoxemia, in which a FiO_2_ > 0.6 may be required to prevent end-organ damage. Additionally, it is not uncommon for pilots, divers, and astronauts to inhale high concentrations of oxygen prior to operations in order to reduce the risk of decompression sickness (Webb and Pilmanis, 1993, 1998; United States Navy, 2011). In these scenarios, lung injury known as pulmonary oxygen toxicity (PO_2_T) may develop.

Pulmonary oxygen toxicity is a progressive disease heralded by acute tracheobronchitis, which manifests as cough and burning sensation with respiration. Findings may progress to include rales on auscultation, atelectasis, non-cardiogenic pulmonary edema, acute parenchymal lung injury, and/or chronic lung injury (Jenkinson, 1993) as well as changes in vital capacity (Jackson, 1985), lung compliance and diffusing capacity. Histologically, PO_2_T is divided into two phases. The first stage is the acute exudative phase characterized by inflammation, edema, hemorrhage, swelling and cellular destruction. The second stage is the chronic proliferative phase as interstitial fibrosis develops and type II alveolar epithelial cells proliferate (Crapo, 1986). While the exudative phase is reversible, recovery from the proliferative phase is incomplete and leaves permanent residual scarring. Unfortunately, by the time current diagnostic modalities in pulmonary function testing detect injury, significant pulmonary damage has already occurred.

Without a direct measure to identify PO_2_T, it is impossible to predict the point at which increasing lung injury is being sustained during oxygen administration. Operationally, oxygen toxicity is prevented by calculating the Unit Pulmonary Toxicity Dose (UPTD) developed at the University of Pennsylvania in 1970 (Bardin and Lambertsen, 1970; Clark and Lambertsen, 1970). A unit dose is the effect of 1 min of exposure at 1 atm PO_2_. The endpoint the UPTD model utilizes is a reduction in lung vital capacity (VC). VC was chosen because it was an objective measure that could be obtained non-invasively. The challenge with using vital capacity is that the daily physiologic variation in VC of 5% is 2.5 fold the median decrease (2%) marked as a safe threshold of exposure (Wingelaar et al., 2019a). Despite attempts to refine the UPTD with other measures such as diffusion capacity, the decrease in VC remains as the key objective measure (Arieli et al., 2002). Finally UPTD is not an individualized measure which correlates poorly to individual disease onset and has never been validated against later stages of PO_2_T such as the exudative phase which involve different pathological endpoints.

Recent human and animal research has demonstrated that prior to the onset of clinical symptoms, hyperoxia induces significant oxidative stress that overwhelms inherent antioxidant enzymes and leads to lipid peroxidation (Deneke and Fanburg, 1980; Freeman and Crapo, 1981). Lipid peroxidation in turn produces volatile organic compounds (VOCs) such as alkanes, isoprenes, and methylated alkanes that can be detected in exhaled breath. More than 3,000 exhaled VOCs have been detected to date, and of these compounds, an estimated 1% are likely disease-specific (Phillips et al., 1999).

A predictive non-invasive monitoring test would be an effective tool for diagnosis and management of many disease states. The pulmonary system is not unique in that desire; as such the non-invasive measurement of VOCs is obviously attractive. VOCs have been applied to multiple lung conditions including acute respiratory distress syndrome (Bos et al., 2014a, b), pulmonary infections (Phillips et al., 2012; Sethi et al., 2013), cancer (Phillips et al., 2003a; Poli et al., 2005; Dent et al., 2013) and asthma. In asthma VOC analysis was able to separate preschool asthmatic children from those with transient wheezing and VOC analysis has been able to predict asthma exacerbation (Sethi et al., 2013). Though not yet applied to management considerations, perhaps VOC based asthma management may someday gain the acceptance that exhaled Nitric Oxide currently enjoys (Phillips et al., 2012).

Volatile organic compound-based biomarker research in the setting of oxygen exposure is an area of research focus (Phillips et al., 2003b; van Ooij et al., 2014; Wingelaar et al., 2019a, b). Biomarkers were detected in as little as 30 min of hyperoxic exposure in otherwise healthy, asymptomatic individuals (Phillips et al., 2003b). Similarly, significant changes in exhaled molecular profiles of five VOCs were observed after submerged oxygen diving (van Ooij et al., 2014). A third study longitudinally measured VOCs at five time points post dive and identified seven compounds after submerged hyperbaric hyperoxic conditions (Wingelaar et al., 2019b). A fourth study demonstrated changes in VOCs associated with hyperbaric oxygen in a dry chamber dive (Wingelaar et al., 2019a), however, that group showed differences in VOC profile from their previous submerged wet dives (Wingelaar et al., 2019b). However, these studies did not demonstrate any correlation between the presence of oxygen-specific VOCs and pulmonary injury. Therefore, the purpose of this study was to evaluate the utility of a VOC-based breath test to detect the onset of PO_2_T using clinically relevant endpoints of PaO_2_/FiO_2_ ratio and histopathologic endpoints of interstitial pulmonary edema in a swine model of hyperoxia exposure.

## Materials and Methods

The experiments reported herein were conducted in compliance with the Animal Welfare Act and per the principles set forth in the “Guide for Care and Use of Laboratory Animals,” Institute of Laboratory Animals Resources, National Research Council, National Academy Press, 2011. Before commencing, the study protocol was reviewed and approved by the Water Reed Army Institute of Research/Naval Medical Research Center Institutional Animal Care and Use Committee in compliance with all applicable Federal regulations governing the protection of animals in research. The institutional animal care facility is fully AAALAC accredited, and the veterinary staff is familiar with the swine model of PO_2_T.

### Animals

Juvenile Male Yorkshire swine between 2 and 3 months of age (*n* = 22; 29.52 ± 3.2 kg; Biotechnical Industries, Dunsborough, PA, United States) were selected for the study. The juvenile age provides a protective bias against pulmonary oxygen toxicity, however, the model has been used successfully in pulmonary oxygen toxicity models (Ichikado et al., 2000). The animals were examined by a veterinarian upon delivery. Animals demonstrating any systemic or localized disease process to include but not limited to a respiratory infection were rejected and not allowed to enter the study. Once accepted, animals were housed in free-running cages at the animal care facility for five days before any procedures. Animals were provided a 12 h light/dark cycle, water *ad libitum*, and twice daily feedings (2–2.5% body weight; Lab Diet Pig Grower, ASAP Animal Specialties and Provisions, Elkridge, MD).

### Surgical Preparation

Swine were anesthetized and underwent vascular access port (VAP) placement and external carotid artery cannulation 96 h prior to the experiment to allow recovery from surgical procedures before exposure. Anesthesia induction was performed with intramuscular injection of ketamine (20 mg/kg; Ketathesia USP Injection 100 mg/mL; Henry Schein Animal Health, Dublin, OH, United States) and xylazine (2 mg/kg; Anased Injection 100 mg/ml; Lloyd Shenandoah, Iowa). After induction, animals were endotracheally intubated and maintained on isoflurane inhalant anesthesia (1–3%; Halocarbon Products, River Edge, NJ, United States). Catheterization was performed using a 5–7 cm cranio-caudal incision in the ventral neck over the left or right carotid artery. Blunt and sharp dissection was used to expose and isolate the carotid artery, which was then cannulated with a 14-french intravascular catheter. Once inserted, the catheter was unclamped to confirm placement and flushed with heparinized saline. For placement of the port, a 5–6 cm curvilinear incision was made just dorsal and cranial to the right or left scapula. Subcutaneous tissues were undermined to create a pocket for the port and the catheter was tunneled between the skin and the subcutaneous tissues and attached to the VAP (Solomon Scientific, Skokie, IL, United States). The port was then secured to the underlying musculature and functionality was confirmed by performing a 3 mL blood draw from the port. Subcutaneous tissues were closed with 2-0 suture and skin incisions were closed with a skin stapler. Functionality of the VAP was again confirmed by performing a 3 mL blood draw. The port was then flushed with normal saline and locked with 1 mL heparin (1000 International Units [IU]/mL). An overlying occlusive bandage was placed over the carotid cut-down site and secured with Elastikon.

### Study Design and Environmental Exposure

Following a four day recovery from VAP surgery, swine were randomized by lot into two treatment groups:

• Air Control Group: (0.209 FiO_2_ for 72 h): (*n* = 12)
• Oxygen Group: (>0.95 FiO_2_ for 72 h): (*n* = 10)

Individual animals were placed in a custom-made Plexiglas run and exposed to room air for three background VOC samples spaced 12 h apart. Immediately following the third VOC sampling, the box atmosphere was switched to the appropriate exposure (room air or oxygen). For safety reasons, researchers and technicians conducting the experiment were unblinded to the exposure, however, researchers conducting the pathologic and VOC analysis were blinded to exposure. Oxygen exposure was considered initiated when the FiO_2_ reached >0.95 within the Plexiglas box. These conditions were maintained for 72 h at normobaric pressure (1 atmosphere [ATM]). Air composition of the Plexiglas runs was monitored with a Gas Analyzer (Alpha Mega 9600, Lincoln, RI, United States) and maintained at a stable temperature of 24°C (±0.22°C), 76.9% (±0.96%) humidity, and <0.05% (±0.05%) carbon dioxide (CO_2_). Animals had free access to food and water throughout the treatment period.

The schedule of sample collection was as follows: for all animals, three baseline samples (breath VOC samples, blood, and physiologic recordings) were collected every 12 h for 36 h with the animal breathing room air prior to randomization. Samples were then collected after 12, 24, 36, 48, 60, and 72 h of treatment exposure.

### Breath VOC Sample Collection

Breath VOC samples were collected in awake, non-sedated swine via a sample tube (Carbotrap-C & Carbopack-B, Supelco, Bellefonte, PA, United States) attached to a nose cone. The method has been described in detail (Phillips et al., 2003a). The breath collection apparatus (Menssana Research, Inc., Fort Lee, NJ, United States) was interfaced with the nose cone in order to collect samples from the animals as previously demonstrated in a separate animal study (Ichikado et al., 2000). To avoid affecting the exhaled breath profile, swine were fed a standard diet from arrival at the facility that did not deviate throughout the study period. Animals were not fed their morning or evening meals until after VOC samples were collected. Prior to sample collection animals were maintained on a breathing circuit via the nose cone supplied with room air or >0.95 FiO_2_ for a minimum of 4 min prior to attaching the sample tube. The solvent trap was then connected for 4 min to collect an exhaled breath volume equivalent to two liters of alveolar breath. A separate background sample of breathing circuit gas was also collected.

Each breath sample and paired background sample were sealed and stored at room temperature. After all study samples were collected, the samples were shipped overnight to Menssana Research, Inc., Breath Research Laboratory (Newark, NJ, United States) where they were held at −15°C until analyzed. Sample processing and analysis are detailed in the statistics section.

### Arterial Blood Gas Analysis

Arterial blood gas samples were collected from the VAP using a 1 mL heparinized arterial blood sample syringe (Smiths Medical ASD, Inc., Keene, NH, United States) connected to a 22 gauge Posi-Grip^TM^ Huber needle (Access technologies, Skokie, IL, United States). Prior to drawing the sample, all swine breathed 100% oxygen via nose cone for 5 min. The sample was then analyzed using an ABL800 flex bench-top blood gas analyzer (Radiometer America Inc., Brea, CA, United States) for PaO_2_ and PaCO_2_ content.

### Physiological Monitoring

Respiration signals were detected using a Rheoencephalogram KR – Ea Rheo Preamp (OTE Galileo, Italy) connected to a PowerLab (ADInstruments) data acquisition device. The Rheoencephalogram consists of an analog channel connected to two electrode cables connected to gel electrodes positioned on either side of the ribcage. Electrocardiogram (ECG) signals were detected using a Bioamplifier (Gould) connected to a PowerLab data acquisition device. The Bioamplifier consists of an analog channel connected to four electrode cables. The electrode cables were connected to four gel electrodes positioned on the left shoulder, right shoulder, left subcostal area, and right subcostal area. Respiration and ECG data were recorded and analyzed in LabChart software on a laptop that connected to the PowerLab.

### Histologic Assessment of Lung Injury

After 72 h the swine was euthanized (Euthasol^®^ 1–1.5 ml/10 lbs) and the lungs excised. The right lung was collected, weighed and placed in an oven at 70°C until no further weight loss. Ratios are presented as a mean of the upper and lower lobes. The left lung was fixed via tracheal instillation at a standard pressure of 25 cm H_2_0 with 10% neutral buffered formalin. The lungs were then sectioned into ten cranial to caudal serial sections, each section approximately one centimeter (cm). Three, 1 cm blocks were cut from each section and post-fixed in 10% neutral buffered formalin, trimmed, embedded in paraffin and cut into 5 μm sections for histological staining. Histology slides were de-paraffinized and stained with hematoxylin and eosin.

For quantitative histologic measurement of interstitial pulmonary edema, bright-field photomicrographs of the stained slides were captured using an Olympus AX80 microscope (Olympus Corporation, Tokyo, Japan) equipped with an 1.25× Olympus plan Apo objective and an Olympus DP70 digital camera. Photomicrographs were saved in.tiff file format for analysis using ImageJ64 (Rasband, W. S., ImageJ, US National Institutes of Health, Bethesda, MD, United States, 1997–2011)^1^. ImageJ analysis was performed by a trained observer blinded to group randomizations. Each lung histology image was opened using the ImageJ program and converted to grayscale. The interstitium was outlined using the polygon tool and the background deleted using the clear outside function. The interstitial space was then quantified using the measure function and divided by the total area of the lung section. A ratio of total interstitial area to total section area was determined. The percentage interstitial area of three sections per lung sample was determined and averaged over 10 lung samples. The percentage area of interstitial edema was derived as the mean from all 30 samples per lung. The mean percentage area of interstitial edema for all of the lungs in each treatment group was compiled into a spreadsheet utilizing Microsoft Excel^®^ (2011).

### Statistical Analysis

#### Physiological Data and Histology

All statistical calculations were performed using statistical software (Graphpad Prism, La Jolla, CA, United States). Data was tested for normality using Shapiro–Wilk normality test. All data was found to pass the normality test thus parametric analyses were used. A student’s *t*-test was used for statistical analysis of continuous variables between two groups. A one-way repeated measures ANOVA was used to compare one factor in three or more groups. A two-way repeated measures ANOVA compared two factors in three or more groups. If an overall significance was found, a *post hoc* comparison was made using the Bonferroni Test. A two-tailed *p*-value < 0.05 was considered statistically significant.

#### VOC Analysis

The analysis methods are well described elsewhere (Phillips et al., 2013). Briefly, carbotrap/carbopack sample tubes containing exhaled breath were thermally desorbed to elute trapped VOCs and then processed via two-dimensional gas chromatography-time of flight-mass spectrometry (GCxGC TOF MS) (LECO Pegasus). Features were selected from each chromatogram using the instrument vendor’s software (LECO ChromaTOF). For each detectable peak, the retention time in each dimension, mass/charge ratio and abundance were calculated and recorded and used for identification. Peaks were considered the same species if the retention time for the first dimension was within 5 s and the retention time for the second dimension was within 0.1 s. The signal to noise ratio threshold was 400 for mass/ion abundance. The retention time data and mass/charge ratios provided tentative identification for each compound using the National Institute of Standards and Technology (NIST) compound library. Chromatogram data from the two-dimensional GC/MS analysis were aligned using a special purpose 2D alignment algorithm (DISCO) (Wang et al., 2010) by Prof. Zhang at Louisville. Prospective biomarkers of oxygen exposure were identified by comparing baseline samples with post-exposure samples in the oxygen-exposed swine, and comparing the post-exposure samples between air and oxygen samples. Candidate compounds with a receiver operating characteristic area under the curve (ROC AUC) score greater than a predetermined cutoff of 0.6 were selected for weighted digital analysis (WDA). Weighted digital analysis was used to narrow down a subset of VOCs predictive of oxygen exposure which were combined using an algorithm based on relative abundance. The output of this algorithm was a Breath Score (BS), which reflected the presence of the VOCs in the breath, weighted by the individual C-statistic (AUC of the ROC curve) for oxygen exposure. To determine whether the novel algorithm was predictive of PO_2_T, BS for individual swine were then correlated with clinical manifestations of PO_2_T defined as a PaO_2_/FiO_2_ ratio of < 350 mmHg. The cutoff of less than 350 mmHg was selected as the occurrence of mild hypoxemia before reaching acute respiratory distress syndrome criteria of less than 300 mmHg.

## Results

### Oxygen Exposure Caused a Significant Decline in Pulmonary Function as Determined by Arterial Blood Gas Analysis and Physiological Monitoring

**FIGURE 1:**
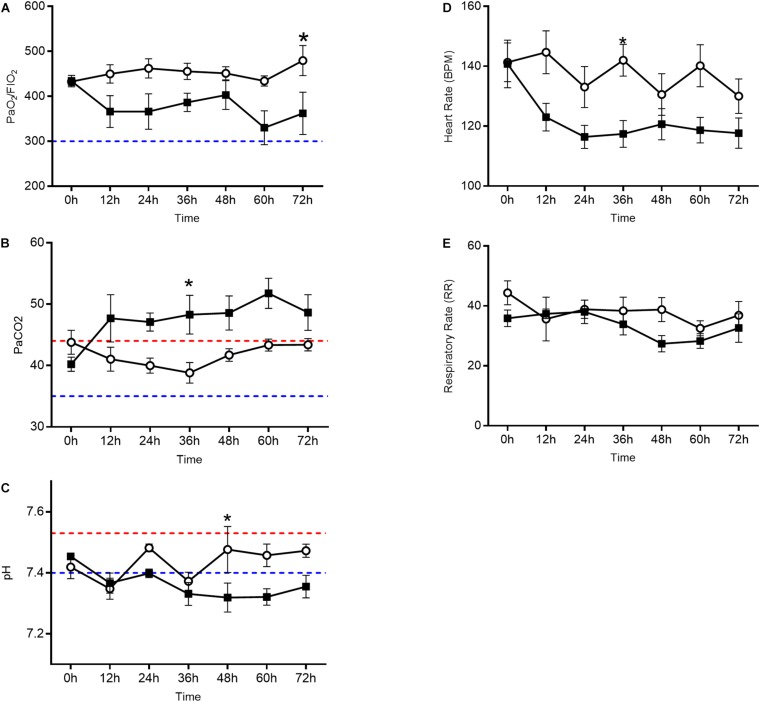
Arterial blood gas analysis and vital sign assessment in the oxygen group (closed squares) compared to the air control group (open circles). Line graphs depict PaO_2_/FiO_2_ ratio **(A)**, PaCO_2_ **(B)**, and pH **(C)** in addition to heart rate **(D)** and respiratory rate **(E)**. The oxygen group had a decreased PaO_2_/FiO_2_ ratio, elevated PaCO_2_, and decreased pH (*p* < 0.0001 repeated measures ANOVA) when compared to the air control group. Each data point represents mean ± SEM. Swine reference ranges for upper (red) or lower (blue) range of normal values are indicated by the dashed lines in **(B)** through **(C)**. For **(A)**, blue dashed line represents threshold for ARDS (Bernard et al., 1994).

Oxygenation as measured by the PaO_2_/FiO_2_ ratio (Figure 1A) decreased in the oxygen group starting at 12 h and each subsequent sample collection time point (*p* = 0.0086). PaCO_2_ (Figure 1B), a surrogate marker for alveolar ventilation, remained elevated in the oxygen group as compared to the air control group at all time points after 12 h (*p* = 0.0099). Blood pH (Figure 1C) decreased in the oxygen group at the 48, 60, and 72 h time points (*p* = 0.0157). The mean heart rate (Figure 1D) was lower in the oxygen group compared to the air group (*p* > 0.05). There was no significant difference in respiratory rate (Figure 1E) between treatment groups.

### Oxygen Exposure Induced Pulmonary Edema

**FIGURE 2:**
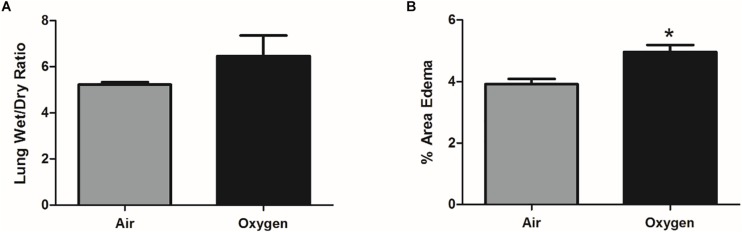
Assessment of pulmonary edema in air control group (gray bar) and oxygen group (black bar). **(A)** Pulmonary edema, quantified by lung wet-dry ratio, showed an increased trend (*p* = 0.07, student’s *t*-test) in the oxygen group when compared to the air control group. Bars represent mean wet-dry ratio ± SEM. **(B)** On direct histological quantification the percent area of interstitial edema was significantly increased in the oxygen group when compared to the air control group. Bars represent mean percent area edema ± SEM. Asterisk represents significance (*p* = 0.0003) as determined by student’s *t*-test.

The wet-dry ratio of the right lung was measured as a gross estimate of pulmonary edema. There was no statistically significant difference in lung weights between groups; however, there was a trend for increased pulmonary edema in the oxygen group (Figure 2A). Interstitial edema was directly quantified from histologic sections of the left lung. The percent area of interstitial edema was significantly increased in the oxygen group as compared to the air group (*p* = 0.0003) (Figure 2B).

### Histopathological Analysis Confirmed That Oxygen-Exposed Swine Developed the Exudative Phase of PO_2_T

Histologic findings in the oxygen group included interstitial edema, congestion and thickening of alveolar septa by inflammatory cells with and without type II pneumocyte hyperplasia (Table 1). Alveolar edema was not a predominant finding in any group at 72 h, however, interstitial edema and dilated lymphatics were common findings in oxygen-exposed animals. Tracheal instillation method may have confounded the alveolar edema findings. Taking together, however, these pathologic changes are consistent with the exudative phase of PO_2_T.

**TABLE 1:** Histological characteristics of the oxygen group.

	**Percent (%)**
**Exudative phase of PO_2_T**	
Acute Neutrophilic Infiltrate	12.5
Interstitial Edema	54.17
Congestion	39.59
Hemorrhage	10.42
**Fibroproliferative phase of PO_2_T**	
Type II Pneumocyte Hyperplasia	4.17

*Values represent the percent of oxygen-exposed swine with the above findings on histology.*

### Analysis of VOC Content in Alveolar Breath Samples Identified 18 Candidate Biomarkers of Oxygen Exposure

**FIGURE 3:**
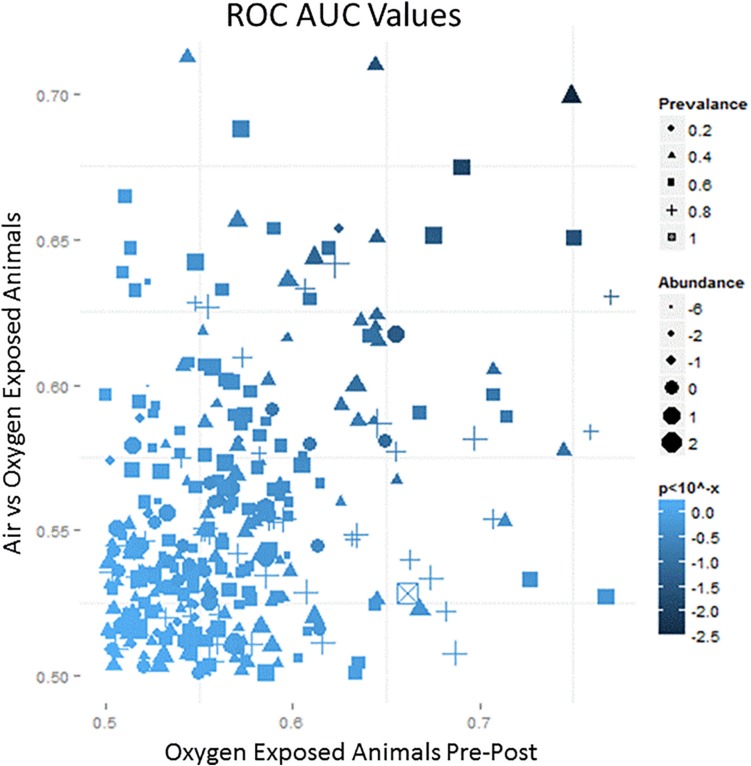
A representative schematic of the VOC content collected from breath samples over the course of the exposure. The *x*-axis represents the ROC/AUC score (probability) that a VOC collected from an oxygen-exposed swine will change from baseline during the exposure period. The *y*-axis represents the ROC/AUC score that a VOC collected from the oxygen-exposed swine during the exposure period will be present in the breath samples collected from air-exposed swine during the exposure period. Symbol shape, area, and shading represent relative prevalence, abundance, and significance, respectively.

Each breath sample produced an average of 700 VOCs with varying abundance. The abundance and prevalence of each VOC peak was aligned across the nine samples from each animal to evaluate changes from baseline. All changes were plotted on an ROC AUC graph with baseline versus exposure samples from oxygen-exposed animals on the x-axis and oxygen-exposed versus room air (control) animals on the y-axis (Figure 3). Thus, VOCs that shifted due to oxygen exposure compared to baseline resulted in a larger *x*-axis value and VOCs that shifted specific to oxygen exposure compared to room air exposure resulted in a larger *y*-axis value. Using a preset cut-off value of 0.625 on the *x*-axis and 0.60 on the *y*-axis, 18 VOCs were identified as candidate biomarkers for detecting oxygen exposure.

### Weighted Digital Analysis Led to the Development of a Predictive Breath Test for Oxygen Exposure Based on Six VOC Compounds

**FIGURE 4:**
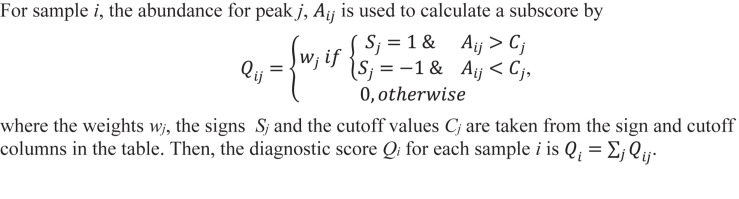
Breath score algorithm derived from the six predictive VOCs using the sign, cutoff, and weighting information in Table 2. *Q*_*ij*_ is the breath score referred to in this work.

**FIGURE 5:**
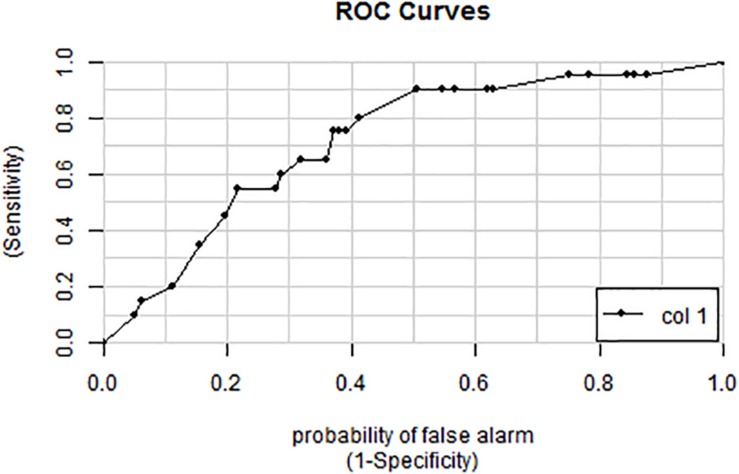
ROC curve for the breath score as a predictor of oxygen exposure. The area under the curve is 0.707 representing a 70.7% probability of predicting oxygen exposure.

The 18 candidate VOCs were then analyzed by weighted digital analysis (WDA) to evaluate changes in abundance from baseline to exposure for both oxygen-exposed and room air-exposed subjects. From the WDA analysis, 6 of the 18 candidate VOCs were specific for oxygen exposure and included: benzene; 2,3,4-trimethyl pentane; 1,4-dimethyl-trans cyclohexane; 2,2,4-trimethyl-hexane; 1,7,7-trimethyl-tricyclo heptane; 4-ethyl-3-octene. Similar to Thrall et al. (2013), an algorithm based on the relative abundance of the six VOCs was developed. Specific peak values Aij were calculated by integrating using the quant-mass m/z in Table 2, and then normalizing the peak area to the area of the internal standard (2 ppm Bromofluorobenzene). Peaks were included if their first retention time (RT1) was within 5 s and second retention time (RT2) was within 0.1 s of the time in Table 2. The output of this algorithm was a breath score, which reflected the presence of the VOCs in the breath, weighted by the individual C-statistic (AUC of the ROC curve) for oxygen exposure (Figure 4 and Table 2). The ROC/AUC curve of the breath score had a value of 0.707, indicating a 70.7% probability of predicting oxygen exposure (Figure 5).

**TABLE 2:** Volatile organic compound algorithm inputs.

**Chemical name**	**m/z**	**RT1 (s)**	**RT2 (s)**	**Weight**	**Sign**	**Cutoff**
Benzene	78	171.30	1.2	1.33	−1	1.10
2,3,4-trimethyl-pentane	71	245.67	1.2	1.45	−1	0.35
1,4- dimethyl-, trans-cyclohexane	97	280.02	1.3	1.26	−1	0.059
2,2,4-trimethyl-hexane	57	285.02	1.2	1.37	−1	0.70
1,7,7-trimethyl-tricyclo[2.2.1.0(2,6)] heptane	93	500.15	1.6	1.28	−1	0.045
4-ethyl-3-octene	69	578.11	1.5	1.27	−1	0.031

*Algorithm inputs of the six predictive VOCs included for calculating the breath score.*

### Breath Score Predicts Onset of PO_2_T as Determined by Arterial Blood Gas Analysis

**FIGURE 6:**
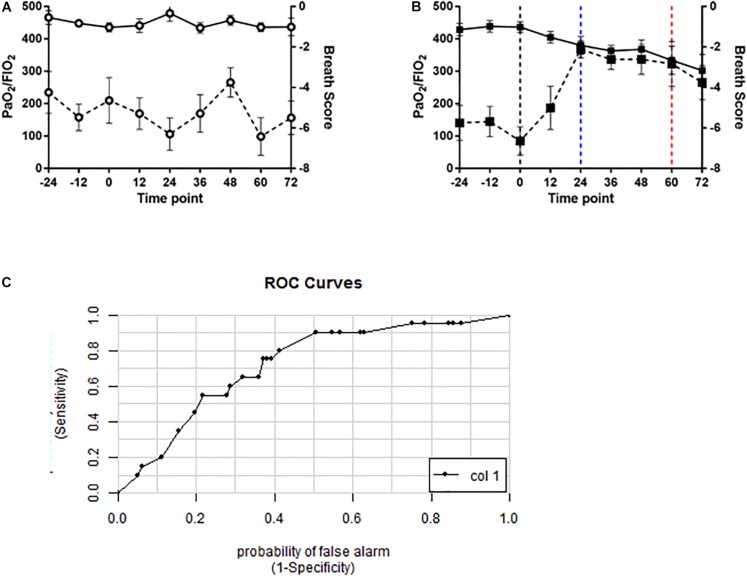
Volatile organic compound based breath score predicts decrements in PaO_2_/FiO_2_ ratio in oxygen-exposed swine. Line graphs depicting breath score (dashed line) and PaO_2_/FiO_2_ ratio (solid line) in **(A)** air controls (open circles) and **(B)** oxygen exposed (closed squares) swine. Vertical black dashed line indicating randomization timepoint. Vertical blue dashed line indicated significantly increased breath score. Vertical red dashed line indicates the P/F ratio < 350 mmHg. Breath scores were significantly increased from baseline in oxygen-exposed but not air controls (*p* < 0.0001, repeated measures ANOVA). **(C)** ROC curve for the breath score predicting a PaO_2_/FiO_2_ ratio of < 350mmHg. The area under the curve is 0.72 indicating a 72% probability that the breath score will predict oxygen toxicity as defined by a PaO_2_/FiO_2_ ratio of 350 mmHg or less.

To determine whether the novel algorithm was predictive of PO_2_T, breath scores for individual swine were then correlated with clinical manifestations of PO_2_T defined as a PaO_2_/FiO_2_ ratio of < 350 mmHg. Mean BS for oxygen-exposed swine (Figure 6B) were significantly increased (*p* < 0.0001) when compared to air-exposed swine (Figure 6A). The BS elevated significantly at the 24 h time point (*p* = 0.0046), and remained significantly elevated until the 72 h time point (*p* = 0.1643). The mean latency between significant BS elevation and P/F ratio decrement cutoff of 350 mmHg was 36 h (mean P/F 334 mmHg at 60 h).

A ROC/AUC curve was generated incorporating the PaO_2_/FiO_2_ ratio and the breath score data (Figure 6C). The predictive value of the breath score to identify animals with PaO_2_/FiO_2_ ratio < 350 mmHg was 0.72.

## Discussion

We identified an exhaled VOC profile that may precede the onset of the exudative phase of PO_2_T, as defined by histologic and functional endpoints. To our knowledge, this finding represents the first pathologically validated sensitive and specific test for PO_2_T. PO_2_T is a limiting factor in the use of oxygen in both military operations and healthcare. Clark (1988) demonstrated that normal human lungs can tolerate an FiO_2_ of 0.5 or less for an unlimited duration of time without the development of prominent oxygen toxicity. Limited studies in human subjects have demonstrated onset of clinical symptoms between 6 and 12 h after exposure to an FiO_2_ of 1.0 (Van De Water et al., 1970), with a change in vital capacity observed after 24 h of exposure and decrements in static compliance and carbon monoxide diffusing capacity seen by 48 h of exposure (Harabin et al., 1987). The maximum safe duration for oxygen exposure between a FiO_2_ of 0.5 and 1.0 is less certain, and the safe upper limit of FiO_2_ for chronic oxygen therapy in an ambulatory setting is undefined. Because early detection of oxygen toxicity has remained elusive and specific therapy is lacking, limiting FiO_2_ to the lowest amount necessary to achieve a PaO_2_ > 55 mmHg or an oxygen saturation > 90% remains the cornerstone of management (Beers, 2008). Additionally, in hyperoxic environments, the military employs a probability-based tool called the UPTD to maintain oxygen exposure within safe limits and decrease the risk of PO_2_T. In both scenarios, the lack of a sensitive and specific test for PO_2_T has significantly limited the optimal use of supplemental oxygen.

To develop a test specific for PO_2_T, we analyzed the VOC content of the exhaled breath and developed a breath score based on six VOCs that changed due to oxygen exposure and was predictive of PO_2_T. We observed that the breath score began to increase after 12 h of exposure to > 0.95 FiO_2_, and reached peak levels at the 24 h time point and remained elevated for the remaining duration of treatment. This pattern was seen in all six VOC compounds and moderately predicted PO_2_T, when defined as a PaO_2_/FiO_2_ ratio less than 350 mmHg. Our predictive value observed for the breath score corroborates previous reports of VOC-based strategies for detecting pulmonary injury (Bos et al., 2014a). We believe the BS precedes evidence of pulmonary injury histopathologically based on a similar exposure profile also in juvenile Yorkshire swine comparing radiographic and histopathologic changes of hyperoxia exposure did not detect changes until 72 h (Ichikado et al., 2000). However, confirmation that the VOC pattern precedes the exudative phase would require a repeat study with both pathologic and VOC analysis at the 12 and 24 h time points.

The advantage to using the GC-MS techniques, as illustrated from our study, is the ability to further identify the individual VOCs, which presents opportunities to further investigate the relationship of individual VOCs to the disease of interest. Of the six VOCs identified, two have been previously identified as components of human breath: benzene (Poli et al., 2005) and 1, 4-dimethyl-trans cyclohexane (Sethi et al., 2013). We did not observe any differences in exhaled pentane between treatment groups, which is similar to the findings reported by van Ooij et al. (2014). In contrast, several other previous studies investigated pentane as exhaled biomarkers of PO_2_T (Morita et al., 1986; Loiseaux-Meunier et al., 2001). It is possible that a subset of PO_2_T-predictive VOCs is co-eluted with pentane, and these compounds can only be resolved using the more sensitive technique of two-dimensional GC-MS, which we applied in this current experiment. Alternatively, because pentane is a very early marker of PO_2_T this effect may have been missed based on our sampling time points. Unlike previous studies that identified changes in breath methylated alkane contours or specific VOCs with oxygen exposure, we were able to identify 18 biomarkers associated with oxygen exposure, as well as six biomarkers specific to PO_2_T. This process allowed us to create an algorithm that generates a breath score, which is correlated with clinical endpoints consistent with the development of PO_2_T.

Limitations for this study include a small sample size, limited applicability of the oxygen exposure model to specific clinical or operational scenarios, and lack of validation in a human study. With the exception of benzene, the VOC biomarkers identified in our study were methylated alkanes, consistent with prior research examining biomarkers from divers exposed to oxygen (van Ooij et al., 2014) and the global changes in methylation seen in total exhaled VOCs following short-duration oxygen exposures in human volunteers (Phillips et al., 2003b). The group of methylated alkanes associated with PO_2_T in our study was unique from those identified as predictive of ARDS (octane, acetaldehyde, and 3-methyl-heptane) (Bos et al., 2014b), indicating that these are not generalized lung injury markers. Interestingly, there was no overlap between the VOCs identified in this study compared with the human diver study conducted by van Ooij et al. (2014). While VOCs represent metabolic changes that should be evolutionarily conserved, there are likely to be subtle interspecies differences in the exhaled VOC profiles between swine and humans, which may partially explain the discrepancy between our findings and the van Ooij et al. (2014) study. Furthermore, the oxygen exposure was of a longer duration in our study (72 h) when compared to the van Ooij et al. (2014) study (1 h), in which there was no evidence of pulmonary function decrements in humans. Additionally, this is further supported by our observation that the breath score peak did not reach a maximum until the 24-h time point, or until decrements in the PaO_2_/FiO_2_ ratio were observed. Thus, the difference in oxygen exposure durations may be the largest contributor to the lack of overlap seen in the VOC biomarkers between our study and the van Ooij et al. (2014) study. Lastly, the experimental setting of this study was normobaric, non-submerged, hyperoxia. Previous work has demonstrated that hyperbaric hyperoxia exposure greatly accelerates pulmonary injury and can be less inflammatory in nature the higher the inspired oxygen pressure (Demchenko et al., 2007). Furthermore, Wingelaar et al. (2019a, b) demonstrated a difference in VOC profiles in their human studies from dry and submerged dives citing increased mechanical load and decrease in pulmonary compliance from high gas densities. All of these factors will need to be accounted for in improving the animal model or with transition of this modality to human operational scenarios.

The ability to identify VOC biomarkers of PO_2_T correlating with decrements in pulmonary function during prolonged hyperoxia exposure has significant potential for applications in clinical and military settings. While our findings demonstrate that exhaled VOCs can predict PO_2_T, additional research is needed to validate and transition this technology into a test with any significant clinical utility. Our study was designed as a biomarker discovery study intended to demonstrate the feasibility of using exhaled VOCs to detect PO_2_T. In particular GC-MS is a time and resource intensive process which is suited for discovery, but has challenges as a point of care device. Further work to confirm and refine this technology to fit specific operational and clinical needs is necessary.

## Data Availability Statement

The datasets generated for this study are available on request to the corresponding author.

## Ethics Statement

The study protocol was reviewed and approved by the Walter Reed Army Institute of Research/Naval Medical Research Center Institutional Animal Care and Use Committee in compliance with all applicable Federal regulations governing the protection of animals in research. The experiments reported herein were conducted in compliance with the Animal Welfare Act and per the principles set forth in the “Guide for Care and Use of Laboratory Animals,” Institute of Laboratory Animals Resources, National Research Council, National Academy Press, 2011.

## Author Contributions

WC, AF, RM, and AH contributed to overall study design, study execution, data analysis, and manuscript preparation. KW and PK contributed to the data analysis and manuscript preparation. RC and MP contributed to the study execution, data analysis, and manuscript preparation.

## Disclaimer

The views expressed in this manuscript are those of the authors and do not necessarily reflect the official policy or position of the Department of the Navy, Department of Defense, nor the U.S. Government. The authors are military service members (or employees of the United States Government). This work was prepared as part of their official duties. Title 17 U.S.C. §105 provides that “Copyright protection under this title is not available for any work of the United States Government.” Title 17 U.S.C. §101 defines a United States Government work as a work prepared by a military service member or employee of the United States Government as part of that person’s official duties.

## Conflict of Interest

PK, RC, and MP were employed by Menssana Research, Inc. The remaining authors declare that the research was conducted in the absence of any commercial or financial relationships that could be construed as a potential conflict of interest.

## Footnotes

1 http://imagej.nih.gov/ij/
